# A new allergic rhinitis therapy (MP29-02*) provides effective and rapid symptom relief for patients who suffer most from the bothersome symptoms of nasal congestion or ocular itch

**DOI:** 10.1186/2045-7022-5-S4-P33

**Published:** 2015-06-26

**Authors:** Wytske Fokkens, Peter Hellings, Joaquim Mullol, Ullrich Munzel, Claus Bachert

**Affiliations:** 1 Academic Medical Center, Amsterdam, Netherlands; 2 University Hospital Leuven, Dept of Otorhinolaryngology, Head & Neck Surgery, Leuven, Belgium; 3 Hospital Clinic IDIBAPS, CIBERES, Barcelona, Spain; 4 Meda, Corporate Clinical Affairs, Bad Homburg, Germany; 5 Ghent University Hospital, Department of Oto-Rhinolaryngology, Ghent, Belgium

## Background

Allergic rhinitis (AR) patients often present with a predominant symptom. Nasal congestion and ocular symptoms have the greatest negative impact on patients’ quality of life [1]. Our aim was to assess the efficacy of MP29-02* (a novel intranasal formulation of azelastine hydrochloride [AZE] and fluticasone propionate [FP] in an advanced delivery system) in seasonal AR (SAR) patients presenting with nasal congestion or ocular itch predominantly compared to AZE, FP or placebo (PLA) nasal sprays.

## Methods

610 patients (≥12 yrs old) with moderate/severe SAR were randomized into a double-blind, PLA-controlled, 14-day, parallel-group trial to MP29-02*, AZE FP or PLA nasal sprays (all 1 spray/nostril bid [total daily doses: AZE 548μg; FP 200μg]). Patients were categorized as nasal congestion- or ocular itch-predominant (for those patients with baseline rTOSS ≥ 8) according to maximal symptom scores at baseline. Targeted symptom reduction was assessed for each predominant symptom over the entire 14 day period and on each day.

## Results

Congestion-predominant MP29-02*-patients experienced 3 times the congestion relief of FP-patients (p=0.0018) and 5 times the relief provided by AZE (p=0.0001). AZE and FP did not significantly differ from PLA. Superior congestion relief afforded by MP29-02* in these patients was evident from Day 2 vs FP (p=0.0155), AZE (p=0.0032) and PLA (p=0.0010) and sustained for 14 days. The level of relief achieved by MP29-02* patients on Day 2 (-0.90) was not achieved before Day 9 by either FP or AZE patients. Ocular itch predominant MP29-02*-patients experienced 4 times the ocular itch relief as FP-patients (p=0.0026) and twice the relief provided by AZE (p=0.0551). FP did not provide additional ocular itch relief over the placebo response. The level of ocular itch relief achieved by MP29-02* patients on Day 2 (-0.93) was not achieved before Day 9 by FP patients or before Day 4 by AZE patients.

## Conclusion

Unlike currently available first line therapy, MP29-02* effectively and rapidly reduced nasal congestion and ocular itch in patients suffering predominantly from these symptoms. MP29-02*’s rapidity and effectiveness in relieving predominant congestion and ocular itch could lead to a reduction in the need for concomitant decongestants and eye drops, respectively and further supports the position of MP29-02* as the drug of choice for the treatment of AR.

* Dymista
